# The anticancer effects of chaetocin are independent of programmed cell death and hypoxia, and are associated with inhibition of endothelial cell proliferation

**DOI:** 10.1038/bjc.2011.522

**Published:** 2011-12-20

**Authors:** C R Isham, J D Tibodeau, A R Bossou, J R Merchan, K C Bible

**Affiliations:** 1 Department of Oncology, Mayo Clinic, 200 First Street SW, Rochester, MN 55905, USA; 2 Sylvester Comprehensive Cancer Center, University of Miami School of Medicine, 1475 NW 12th Avenue (D-1), Miami, FL 33136, USA

**Keywords:** thioredoxin, reactive oxygen species, apoptosis, hypoxia, angiogenesis

## Abstract

**Background:**

We previously reported that chaetocin has potent and selective anti-myeloma activity attributable to reactive oxygen species (ROS) induction imposed by inhibition of the redox enzyme thioredoxin reductase; we now detail its effects in solid tumours.

**Methods:**

Cellular assays, transcriptional profiling and the NCI60 screen were used to assess the effects of chaetocin in solid tumour and endothelial cells.

**Results:**

NCI-60 screening demonstrated chaetocin to even more potently inhibit proliferation in solid tumour than in haematological cell lines; transcriptional profiling revealed a signature consistent with induction of inflammatory response and cell death pathways. Chaetocin induced ROS, oxidative damage to cellular proteins and apoptosis, with 2–10 nM IC_50_s (24 h exposures) in all tested solid tumour cell lines. The pan-caspase inhibitor zVAD-fmk did not block chaetocin-induced cell death despite inhibiting mitochondrial membrane depolarisation and apoptosis. Further, Molt-4 rho^0^ cells lacking metabolically functional mitochondria were readily killed by chaetocin; in addition chaetocin-induced cytotoxicity was unaffected by autophagy inhibitors or hypoxia and consequent HIF-1α upregulation. Moreover, chaetocin inhibited SKOV3 ovarian cancer xenografts producing less vascular tumours, and inhibited human umbilical vein endothelial cell proliferation.

**Conclusion:**

Chaetocin has intriguing and wide-ranging *in vitro* and *in vivo* anticancer effects, and is an attractive candidate for further preclinical and clinical development.

Chaetocin is a natural product produced by *Chaetomium* spp. and related fungi (Sekita *et al*, 1981; Freire *et al*, 2000) with its structure elucidated in 1970 (Hauser *et al*, 1970) indicating an unusual bridged disulphide diketopiperazine core. We became interested in chaetocin initially because of its unique chemical structure, discovering that it has potent and selective *in vitro*, *ex vivo* and *in vivo* anti-myeloma activity attributable to its ability to impose cellular oxidative stress (Isham *et al*, 2007). Subsequently, we discovered that chaetocin serves as a competitive substrate (with respect to thioredoxin) and an inhibitor of the redox enzyme thioredoxin reductase, apparently thereby accounting for its ability to induce cellular oxidative stress and kill tumour cells, as we were unable to define other effects of chaetocin on reactive oxygen species (ROS) or ROS remediation systems to otherwise account for observed effects (Tibodeau *et al*, 2009). Chaetocin has also been found by others to inhibit histone methyltransferase SU(VAR)3–9 (Greiner *et al*, 2005) and HIF-1a signalling (Kung *et al*, 2004; Lee *et al*, 2011)—with interest in the compound sufficient to prompt its recent total synthesis (Iwasa *et al*, 2010; Kim and Movassaghi, 2010). As chaetocin serves as a potent inducer of cellular ROS, apparently consequent to its ability to inhibit thioredoxin reductase-mediated ROS remediation, we postulated that chaetocin might not only have activity in myeloma, but also in solid tumours, as the imposition of cellular ROS has been postulated to represent an attractive candidate therapeutic strategy in solid tumours (Fang *et al*, 2007). We had initially anticipated that its effects might be greatest in haematological tumours, but NCI-60 screening unexpectedly indicated chaetocin to be even more potent in solid tumours, prompting the present more extensive studies.

## Materials and methods

### NCI-60 assay

Chaetocin (Sigma, St Louis, MO, USA) was supplied to the National Cancer Institute for evaluation in the 60 human tumour cell line tumour panel, which they performed as previously described (Monks *et al*, 1991).

### Transcriptional profiling

Total RNA was purified from both A549 and OCI-MY-5 cells treated with chaetocin for 24 h using TRIzol (Invitrogen, Carlsbad, CA, USA) reagent followed by RNeasy kit (Qiagen, Valencia, CA, USA). The resulting purified RNA was submitted to the Advanced Genomic Technology Centre Microarray Shared Resource at Mayo Clinic for RNA integrity analysis on the Agilent 2100 Bioanalyzer (Agilent Technologies, Palo Alto, CA, USA), reverse transcription, labelling and hybridisation to Affymetrix human U133 Plus 2.0 arrays (Affymetrix, Santa Clara, CA, USA). Affymetrix arrays were scanned with the GeneChip 3000 Scanner (Affymetrix). Significant data sets were generated using GeneSpring (Agilent Technologies) and by our statisticians. Common top pathways were generated from the data sets using GeneGo (St Joseph, MI, USA).

### Tissue culture

Cells were cultured in the following media: A549, U2OS, HCT116, and HeLa in RPMI 1640 containing 5% FBS; OCI-MY5 in RPMI 1640 containing 10% FBS; PC-3 cells in F12 medium containing 10% FBS; SKOV3 in McCoy's 5a containing 10% FBS; HEPG2 in Dulbecco's modified Eagle's Medium (DMEM) containing 10% FBS and MCF7 cells in Iscove's modified essential medium containing 5% FBS. All media contained 100 U ml^−1^ penicillin G and 100 *μ*g ml^−1^ streptomycin. Cell lines were passaged twice weekly and maintained at 37 °C (95% air, 5% CO_2_). For hypoxia experiments, HEPG2 and SKOV3 cells were maintained at 37 °C instead in an atmosphere containing 94% N_2_, 1% O_2_ and 5% CO_2_. OCI-MY5 cells were kindly provided by Dr Diane Jelinek (Mayo Clinic, Rochester, MN, USA). All other cell lines were originally obtained from ATCC (Chicago, IL, USA).

### Generation of cell lines lacking mitochondrial DNA (mtDNA)

MOLT-4 cells lacking mtDNA were produced by culture in DMEM containing 4500 mg l^−1^ glucose and 1 mM pyruvate supplemented with 5% FBS, 50 *μ*g ml^−1^ uridine, and 50 ng ml^−1^ ethidium bromide. Cells are allowed to grow for 3–4 weeks in the presence of ethidium bromide in order to isolate cells that completely lack mtDNA. After the confirmation of mtDNA depletion (by either withdrawing uridine from the medium and observing cell death, by western blotting of proteins, including COX I and COX II, or by measuring the oxygen consumption of selected versus parental cells), cells were maintained in DMEM supplemented with 5% FBS and 50 *μ*g ml^−1^ uridine so as to retain the mtDNA-dead phenotype.

### Colony formation assays

Briefly, 500 cells obtained by trypsinisation from stock flasks of subconfluent cells were plated into each of the triplicate sets of 35 mm tissue culture plates and allowed to adhere overnight. Cells were then treated for the indicated times with diluent, chaetocin or as otherwise specified. Where indicated, cells were preincubated with *N*-acetyl cysteine (NAC; Sigma) 30 min before drug addition. After drug removal and washing twice with media, cells were allowed to proliferate in a drug-free medium for 7–10 days; thereafter plates were washed, stained with Coomassie blue, and colonies were counted on an imager using GeneTools software (Syngene, Frederick, MD, USA).

### Assessment of cellular ROS levels

Cellular oxidative stress was assessed utilising 5,6-chloromethyl-2′,7′-dichlorodihydrofluorescein diacetate (CM-DCF) as a cell permeable fluorescent probe with FACS analysis. Briefly, cells were treated with the indicated drug for 24 h, the media was removed, and the cells were then incubated in warm PBS containing 6 *μ*M probe at 37 °C for 1 h. The probe was then removed and warm media was added back to the cells for 10 min. The cells were then trypsinised, sedimented, and resuspended in cold PBS before flow microfluorimetry using a FACScan flow cytometer (Becton Dickinson, Mountain View, CA, USA) with a 488 nm laser. Fluorescence emission was observed through a 530/30 nm filter, and 20 000 events were analysed using CellQuest software (Verity Software House, Topsham, ME, USA). In separate experiments, the tested compounds were found not to directly interact with the probe *in vitro*, as no fluorescence emission was observed at a 1 : 10 dye:compound ratio.

### Oxyblot analyses

Protein lysates were prepared in CelLytic buffer (Sigma) containing 50 mM DTT from HeLa cells treated with various doses of chaetocin for 24 h, with a 30 min preincubation with NAC where indicated. Protein concentrations of the lysates were measured by BCA assay (Thermo Fisher Scientific Inc., Rockford, IL, USA) and 20 *μ*g of protein was used for the OxyBlot Protein Oxidation Detection assay (Chemicon International, Temecula, CA, USA). Samples were denatured with 6% SDS incubated with the provided DNPH solution to derivatise any carbonyl groups for 15 min, and the reaction was stopped with the provided neutralisation solution. The samples were then run on a 10% Bis-Tris SDS–PAGE gel and transferred to a nitrocellulose membrane. Resulting membranes were blocked for 1 h in 1% BSA, then incubated for 1 h with primary antibody specific to DNP residues, and then incubated with an HRP-conjugated secondary antibody for 1 h. Pierce West Pico chemiluminescent detection reagent (Thermo Fisher Scientific Inc.) was added and membranes were exposed to the film.

### Electron microscopy

Cells for transmission electron microscopy were released by trypsinisation, washed twice with PBS, fixed for 1 h with Trump's fixative (1% glutaraldehyde and 4% formaldehyde in 0.1M phosphate buffer (pH 7.2)), treated with phosphate-buffered 1% 0504, stained *en bloc* with 2% uranyl acetate for 30 min at 60 °C and embedded in Spurr's resin. Sections (90 nm) were cut on a Reichert Ultracut E or S Ultramicrotome (Leica, Inc., Vienna, Austria), collected on 200-mesh copper grids, stained with lead citrate, and examined and photographed with a JOEL 1200 EXII electron microscope (Tokyo, Japan) operating at 60 kV.

### Hoechst 33258 staining

Harvested and washed cell suspensions fixed with methanol/acetic acid (3 : 1) were mixed in equal parts with 1 *μ*g ml^−1^ Hoechst 33 258 (Sigma) solubilised in 50% glycerol and 50 mM Tris pH 7, with the induction of apoptosis assessed using fluorescence microscopy by examining cells for apoptotic morphological changes, expressing the number of apoptotic cells at a percentage of 200 total counted cells.

### Evaluation of loss of mitochondrial membrane potential (*ΔΨ*_m_)

Chaetocin-treated A549 cells were sedimented, washed with PBS, resuspended in medium, stained with TMRM (Tetramethylrhodamine methyl ester, Invitrogen; 50 nM, 45 min, 37 °C), placed on ice for 5 min and immediately subjected to flow microfluorometry using a FACScan flow cytometer (Becton Dickinson, 488 nm laser). Fluorescence emission was observed through a 585/42 nm filter, and 20 000 events were analysed using CellQuest software (Verity Software House).

### Immunoblotting

After maintaining and treating the cells as indicated, cold PBS was immediately added, plates were washed twice in cold PBS and adherent cells were lysed in CelLytic lysis buffer (Sigma) containing protease inhibitors (Roche, Indianapolis, IN, USA). As HIF1*α* is subject to nuclear translocation, samples were sonicated for all experiments assessing HIF1α. Total cellular protein was analysed by BCA assay to facilitate equal sample loading, and lysates were electrophoresed on 7.5% SDS–PAGE gels and transferred onto nitrocellulose. Immunoblotting for HIF1*α* (Abcam, Cambridge, MA, USA), vascular endothelial growth factor receptor (VEGFR-1; Cell Signaling), phosphorylated VEGFR-1 (anti-phospho-Flt-1 Tyr1213; Millipore, Temecula, CA, USA) and actin were then performed.

### Xenograft studies

Mouse experiments followed institutional guidelines and were approved by the Institutional Animal Care and Use Committee (IACUC). Briefly, SKOV3 cells were harvested, washed twice and resuspended in PBS (2.5 million cells per 100 *μ*L). Female athymic nu/nu mice (5–6 weeks old) purchased from Harlan Laboratories, Inc. (Indianapolis, IN, USA) were anaesthetised using isoflurane, and were injected subcutaneously in the flank with 100 *μ*l of SKOV3 inoculum. After measurable 4–5 mm tumours were formed, the mice were randomly assigned to control or experimental groups: 0.2 mg kg^−1^ IP 5 × per week of chaetocin (in 0.17% DMSO, 20% polyethylene glycol 400; remainder 0.9% NaCl). Tumours were measured with calipers three times weekly, and tumour volumes were calculated using the formula: volume=A × B × B/2, (where A was the longest tumour dimension and B the smallest).

### Human umbilical vein endothelial cell (HuVEC) studies

A total of 4 × 10^3^ HuVECs (Lonza, Walkersville, MD, USA) in 100 *μ*l medium with 1% FBS, penicillin (100 U ml^−1^), and streptomycin (100 *μ*g ml^−1^) was placed into each well of a 96-well plate, treated with chaetocin at indicated concentrations, and incubated at 37 °C for 72 h. Either *β*FGF (PeproTech, Rocky Hill, NJ, USA; 20 ng ml^−1^) or EGM-2 medium (containing hEGF, VEGF, hFGF-B, R3-IGF-1; Lonza) was used to induce HuVEC proliferation as indicated. After the 72-h incubation, WST-1 (10 *μ*l; Roche, Mannheim, Germany) was added to each well, and after a 3-h incubation at 37 °C, absorbance at 450 nm was determined for each well with a microplate reader (Bio-Rad Laboratories, Hercules, CA, USA). Data presented are the average of triplicate experiments.

## Results

### Chaetocin potently inhibits proliferation and colony formation in a broad range of cancer cell lines

**Figure 1:**
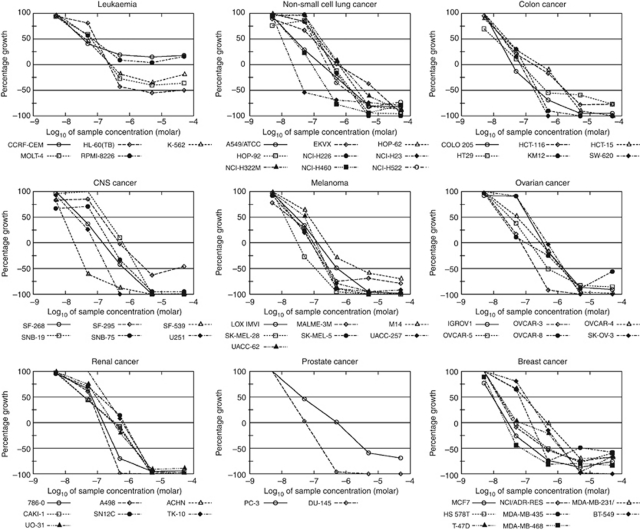
NCI-60 growth inhibition data for chaetocin. Growth inhibition data using high-throughput screening assessing the effects of 48-h continuous chaetocin exposure in 60 human tumour cell lines are graphically represented over broad chaetocin concentration ranges. Note that the inhibition of haematological cell line growth by chaetocin (upper left panel) was much less pronounced in comparison with all tested solid tumour cell lines.

**Figure 2:**
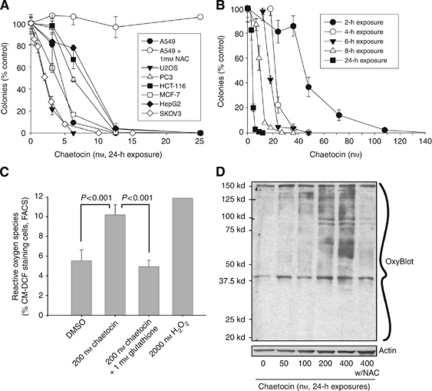
Chaetocin has broad-spectrum anti-neoplastic activity in an array of solid tumour cell lines associated with the induction of cellular oxidative stress and oxidative damage to cellular proteins. (**A**) Inhibition of colony formation by chaetocin in seven solid tumour cell lines. All drug exposures were of 24 h duration, effects of 1 mM NAC pretreatment on the inhibitory effects of chaetocin in A549 cells are also shown. (**B**) Time dependence of the inhibitory effects of chaetocin in A549 cells (colony formation assays, A549 non-small cell carcinoma cells). (**C**) Effects of 200 nM chaetocin on ROS production in OCI-MY5 cells (assessed by FACS, CM-DCF staining). Effects of H_2_O_2_ are shown as a positive control; reversal of ROS induction by 1 mM glutathione also shown. (**D**) Chaetocin treatment of HeLa cells results in the dose-dependant induction of broad-spectrum oxidative damage to cellular proteins (OxyBlot, ability of pretreatment with 1 mM NAC to prevent chaetocin-induced oxidative damage also shown).

In preliminary studies, we examined the effects of chaetocin on cancer cell proliferation using the NCI-60 screen (Monks *et al*, 1991). Although we had previously found chaetocin to have potent anti-myeloma activity, interestingly, NCI-60 results indicated even more potent inhibition of solid tumour than haematological cell line proliferation by chaetocin (Figure 1). Follow-up colony formation assays confirmed the potent antineoplastic effects of chaetocin in a wide range of solid tumour cell lines, with 2–10 nM IC_50_s (24 h exposure) in all assayed lines (Figure 2A). As short as 4 h chaetocin exposure produced near maximal growth-inhibitory effects in A549 cells (Figure 2B).

### Chaetocin induces cellular oxidative stress associated with oxidation of cellular proteins and cell death induction that can be blocked by NAC

Consistent with prior observations in myeloma cells and cell lines (Isham *et al*, 2007) glutathione (GSH) or its cell permeable precursor NACdramatically attenuated chaetocin-induced (i) reduction in colony formation (Figure 2A), (ii) induction of ROS (Figure 2C), and (iii) oxidation of cellular proteins (Figure 2D) without either GSH or NAC directly reducing chaetocin itself (Tibodeau *et al*, 2009), implicating the induction of cellular oxidative stress/ROS by chaetocin as critical to its observed *in vitro* solid tumour antineoplastic activity.

### Chaetocin has pleiotropic effects on cellular metabolism as assessed via transcriptional profiling

**Figure 3:**
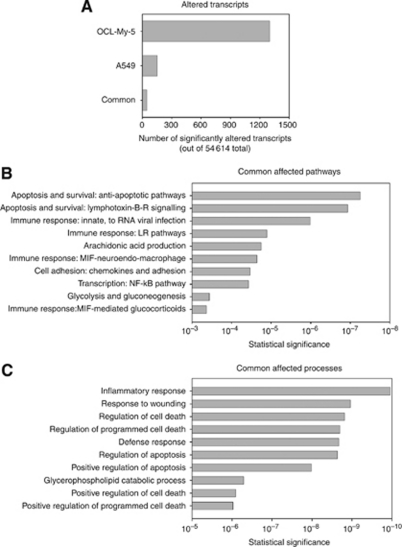
Altered transcripts from transcriptional profiling performed in both OCI-MY5 and A549. (**A**) Analyses performed in GeneGo show the significantly altered transcripts in each cell line and those in common. (**B** and **C**) Further analyses grouped the commonly altered transcripts into top altered pathways and biological processes affected by 100 nM chaetocin (24 h).

As one tool to further examine the cellular effects of chaetocin in solid tumours, we utilised transcriptional profiling targeting identification of transcripts consistently altered by chaetocin across both myeloma and solid tumour cell lines. Interestingly, when comparing the transcriptional profiling results attained in response to identical chaetocin exposures (100 nM × 24 h, Affymetrix platform) in the A549 non-small cell lung cancer *vs* the OCI-MY5 myeloma cell line, it was notable that many more significantly altered transcripts arose in OCI-MY5 compared with A549 cells (Figure 3A). Of the two results, only 48 transcripts were similarly and significantly altered in both cell lines (Table 1); these were analysed using GeneGo software to help elaborate pathways and biological processes altered by chaetocin treatment over control diluent/DMSO treatment (Figure 3B and C). Transcripts related to inflammatory response and cell death/apoptosis pathways were most commonly altered, presumably indicating cellular response to chaetocin-induced oxidative stress as well as compensatory survival signalling pathway activation.

### Chaetocin induces complex morphological changes in association with cell death

**Figure 4:**
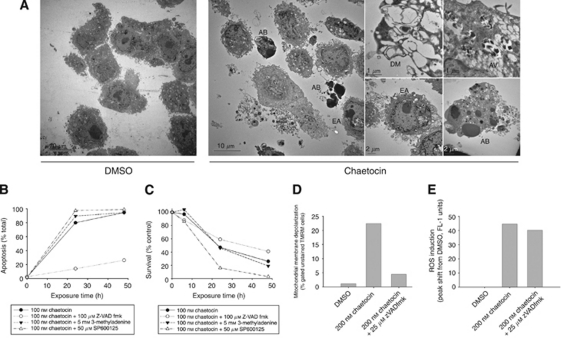
Effects of chaetocin on cell morphology, apoptosis, and mitochondrial membrane depolarisation. (**A**) Effects of chaetocin on A549 cell morphology as assessed by transmission electron microscopy (400 nM, 24-h exposure) EA cells showing nuclear condensation on the periphery as well as late apoptotic cells showing AB. Evidence of autophagy in enlarged panels showing DM and filled AV. (**B**) Chaetocin-induced apoptosis is attenuated by co-treatment with the pan-caspase inhibitor zVADfmk, but not by the autophagy inhibitors 3-methyladenine or SP600125 (assessed by Hoechst 33 258 morphological evaluations, fluorescence microscopy). (**C**) Chaetocin-induced cell death is not blocked by the pan-caspase inhibitor zVADfmk or the autophagy inhibitors 3-methyladenine or SP600125 (assessed by trypan blue exclusion assay). (**D**) Chaetocin-induced mitochondrial membrane depolarisation (assessed by TMRM staining, FACS), but not chaetocin-induced ROS induction (CM-DCF staining, FACS; **E**) is attenuated by the pan-caspase inhibitor zVADfmk.

Electron microscopy demonstrated the induction of complex structural changes in A549 cells treated with chaetocin (400 nM, 24 h exposure) that included apoptotic morphology (chromatin condensation on the nuclear periphery in early apoptotic cells (EA) and pro-apoptotic bodies (AB) in late apoptotic cells), as well as occasional double membrane vacuole (DM) formation and filled autophagic vacuoles (AV), indicating autophagy (Figure 4A). These results raised questions as to on which cell death pathway(s) may chaetocin cytotoxicity depend, prompting further and more detailed experiments.

### Chaetocin-induced mitochondrial membrane depolarisation and apoptosis, but not ROS induction or cytotoxicity, are attenuated by the pan-caspase inhibitor zVAD-fmk

In order to study whether chaetocin-induced ROS induction and cell death might be dependant upon intact apoptotic pathways, experiments were next carried out using an apoptosis inhibitor. Although, as expected, apoptosis was dramatically reduced by co-treatment with the pan-caspase inhibitor zVAD-fmk (Figure 4B); chaetocin-induced cell death was only minimally affected (Figure 4C). In parallel, zVAD-fmk also greatly attenuated mitochondrial membrane depolarisation induced by chaetocin (Figure 4D), but not ROS induction (Figure 4E), altogether indicating that chaetocin-induced cellular oxidative stress and cell death are largely independent of apoptosis induction. This is consistent with the hypothesis that chaetocin induces cellular ROS in a manner independent of mitochondrial ROS release occurring in response to mitochondrial membrane depolarisation in conjunction with the induction of apoptosis.

### Chaetocin-induced cytotoxicity is not inhibited by autophagy inhibitors

As chaetocin treatment led to the formation of both single and bimembrane vacuoles in A549 cells (Figure 4A), suggesting that autophagic pathways might be induced, we also examined the effects of the autophagy inhibitors 3-methyladenine or SP600125 on chaetocin-induced cell death in A549 cells. Neither the PI3 kinase and autophagic inhibitor 3-methyladenine nor the Jun kinase and autophagic inhibitor SP600125 attenuated chaetocin-induced cell death or apoptosis (Figure 4B and C); furthermore, we observed no discernible alterations in MAP-LC3 or Beclin-1 protein level in response to chaetocin treatment (data not shown). Collectively these data indicate that the induction of autophagy, as well as apoptosis, appear not required for the *in vitro* antineoplastic effects of chaetocin.

### Chaetocin-induced ROS production and cell death do not require metabolically functional mitochondria

**Figure 5:**
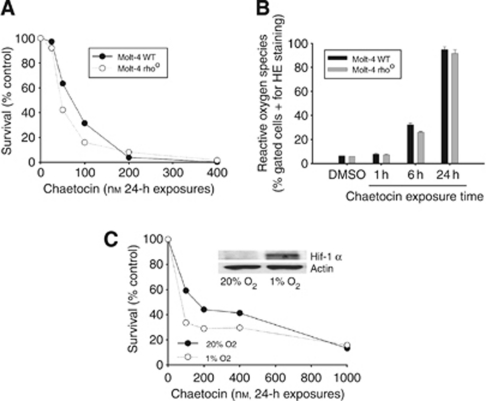
Neither dysfunctional mitochondria nor hypoxia attenuate chaetocin-induced cytotoxicity. (**A**) Chaetocin-induced cytotoxicity (assessed by trypan blue exclusion assay, 24-h chaetocin exposures) is unaltered in Molt-4 rho° cells lacking respiratorily functional mitochondria. (**B**) Chaetocin-induced increases in ROS (assessed by CM-DCF staining, FACS; 24-h chaetocin exposures) is unaltered in Molt-4 rho° cells lacking respiratorily functional mitochondria. Error bars indicate one sample s.d. (**C**) Chaetocin-induced cytotoxicity is not attenuated in response to hypoxic (*vs* normoxic) tissue culture conditions (assessed by trypan blue exclusion assay, 20% *vs* 1% O_2_). Inset shows corresponding HIF-1*α* induction resulting from hypoxia (assessed by SDS–PAGE and immunoblotting).

As it is known that mitochondria are a major source of cellular ROS, and as chaetocin potently induces ROS (Figure 2C) and was observed to induce mitochondrial membrane depolarisation (Figure 4D), we also examined whether chaetocin-induced cell death and/or ROS induction might be dependant upon metabolically functional mitochondria. Neither the cytotoxic effects nor the induction of ROS by chaetocin differed in response to treatment of parental Molt-4 or Molt-4 rho^0^ cells with metabolically inactive mitochondria (Figure 5A and B), bolstering other data (Figure 4C and E) in support of the hypothesis that the ability of chaetocin to induce ROS and cell death *in vitro* appear not to require metabolic ROS production or release from mitochondria despite the ability of chaetocin to induce mitochondrial membrane depolarisation and apoptosis (Figure 4A, B and D). These data are consistent with our prior report indicating that chaetocin does not itself directly induce oxidative stress, but instead apparently triggers cellular ROS accumulation by inhibition of the redox enzyme thioredoxin reductase (Tibodeau *et al*, 2009).

### Chaetocin-induced cell death is slightly enhanced, not attenuated, in parallel with hypoxia-stimulated upregulation of HIF-1*α*

As chaetocin-induced cell death is dependant upon its ability to induce the production of cellular oxidative species (e.g., Figure 2), we became concerned that such effects might be attenuated under hypoxic conditions whereby oxygen tension was reduced and HIF-1*α* was induced. This concern turned out to be unfounded, as we observed slightly enhanced, not attenuated, induction of cell death by chaetocin under *in vitro* hypoxic conditions whereby HIF-1*α* was potently induced (Figure 5C). In addition, of interest is that *in vitro* cytotoxicity of chaetocin was observed to be robust even in the absence of detectable HIF-1*α* (Figure 5C), seemingly in deference to a prior report indicating that chaetocin-induced antitumour effects are dependant upon the presence of functional HIF-1α (Lee *et al*, 2011).

### Chaetocin attenuates the growth of SKOV3 ovarian cancer xenografts in nude mice

As chaetocin demonstrated potent *in vitro* antineoplastic effects in solid tumour cell lines not attenuated by hypoxia-stimulated HIF-1*α* induction, we hypothesised that chaetocin might also have *in vivo* antineoplastic effects in solid tumours, akin to those observed in myeloma (Isham *et al*, 2007). Although we had previously found chaetocin at low dosages to be well tolerated when intraperitoneally administered in mice (Isham *et al*, 2007), in preparation for the present *in vivo* experiments, we first carefully assessed the potential deleterious effects of chaetocin in animals not bearing tumours when treated with escalating chaetocin doses and administration schedules.

To establish the maximally tolerated dosage in response to intraperitoneal chaetocin administration, an IACUC-approved approach akin to that used in human dosage-escalation studies was applied, incrementing doses and the frequency of intraperitoneally administered chaetocin in a stepwise manner so as to gradually increase chaetocin dose-density and enhance the detection of early signs of toxicities. The only observed toxicities from the administration of higher chaetocin dose-density in mice related to evidence of peritoneal irritation associated with IP administration (adhesions, and upon occasion bloody ascites); no evidence of adverse effects of chaetocin treatment on solid organs was noted, and observed peritoneal changes were not observed to have influenced animal feeding behaviour or activity level. Observed peritoneal irritation seemed related primarily to the quantity of chaetocin administered with each IP injection; this accounts for our shift to a 5 × per week administration schedule, allowing us to define the maximally tolerated IP dosing as 0.2 mg kg^−1^ 5 × per week in mice.

**Figure 6:**
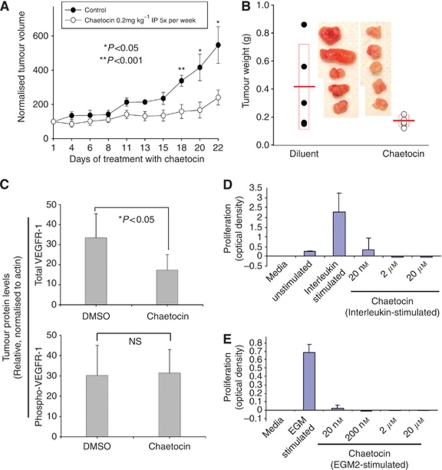
Chaetocin inhibits the growth of SKOV3 xenografts and proliferation of HuVEC. (**A**) Chaetocin treatment (0.2 mg kg^−1^ 5 × per week IP) attenuates the growth of SKOV3 xenografted tumours in nude mice. Tumour volumes calculated on the basis of bi-dimensional tumour measurements using calipers; error bars indicate ±1 sample s.d. (**B**) Chaetocin treatment (0.2 mg kg^−1^ 5 × per week IP) leads to lower weights and lessened vascularity in excised SKOV3 tumours. Lines indicate sample means, boxes indicate±1 sample s.d. (**C**) Chaetocin treatment (0.2 mg kg^−1^ 5 × per week IP) leads to attenuated total, but unaltered phospho-, VEGFR-1 levels in excised SKOV3 tumours lysates. Error bars indicate one sample s.e.m. Chaetocin inhibits interleukin-induced (**D**) or EGM2-induced (**E**) proliferation of HUVECs. Error bars indicate one sample s.d.

Compared with diluent control treatment, chaetocin treatment (0.2 mg kg^−1^ IP 5 × per week) significantly delayed the growth of established SKOV3 tumours (Figure 6A), with minimal evidence of toxicities observed in treated animals. We noted in parallel that excised tumours from chaetocin-treated animals were less vascular in comparison with diluent control tumours (Figure 6B), raising the question of whether anti-angiogenic effects might be in part contributory to observed *in vivo* efficacy, and leading to additional experiments.

### Total VEGFR-1, but not phopsho-VEGFR-1, levels are attenuated in chaetocin-treated SKOV3 xenografts

Seeking a potential explanation for why chaetocin-treated tumours were seemingly less vascular, and realising that phospho-VEGFR-1 level would be expected to represent a surrogate marker for the extent of VEGF activity in xenograft tumours, we examined tumour levels of both total and phospho-VEGFR-1 in homogenate of excised xenografts. Although the mean tumour total VEGFR-1 protein level (assessed by immunoblotting of tumour lysates normalised to paired tumour actin levels) was significantly reduced in chaetocin-treated tumours relative to diluent-treated controls, the corresponding mean phospho-VEGFR-1 level did not differ between the groups (Figure 6C), suggesting that the observed differences in tumour vascularity were not likely attributable to differential VEGFR-1 activation. In parallel, we also examined HIF-1*α* protein levels in treated *vs* control tumour lysates and additionally in treated *vs* control SKOV3 cells *in vitro*; in each instance HIF-1*α* was barely detectable, providing no indication that HIF-1*α* was required for the antitumour effects of chaetocin *in vitro* or *in vivo* in the utilised SKOV3 model systems.

### Chaetocin inhibits interleukin or EGM2-stimulated proliferation of HuVECs

Seeking an alternative potential explanation for the attenuated vascularity observed in SKOV3 tumours excised from chaetocin-treated animals, we went on to examin the effects of chaetocin on the proliferation of HuVECs. Chaetocin potently attenuated HuVEC proliferation stimulated by either interleukin or EGM2 endothelial cell growth medium (Figure 6D and E), suggesting that the anti-proliferative effects of chaetocin on tumour microvasculature might alternatively be a contributor to the observed attenuated vascularity and *in vivo* effects of chaetocin (Figure 6A and B), and raising the intriguing possibility that chaetocin may function *in vivo* not only by targeting tumour directly, but perhaps also in part by simultaneously targeting endothelial cells in the tumour microenvironment.

## Discussion

Presented results importantly demonstrate that chaetocin has substantial activity in solid tumour models and not just in multiple myeloma as we had previously reported. It is particularly intriguing that the antineoplastic effects of chaetocin in solid tumour cell lines (assessed in the NCI-60 screen, Figure 1) are in fact almost uniformly superior to those seen in multiple myeloma and other haematological tumours; this was unanticipated. Moreover, it is noteworthy that observed solid tumour activity translates *in vivo*, as chaetocin potently inhibited SKOV3 ovarian cancer xenografts (Figure 6A and B). Consequently, chaetocin would seem to represent at least as promising a candidate that is therapeutic in solid tumours as in myeloma.

Present studies also add important knowledge related to the effects of chaetocin in relation to programmed cell death (PCD) pathways in solid tumours. As in multiple myeloma, chaetocin potently induces cellular oxidative stress/ROS in solid tumour cell lines that is required for *in vitro* antineoplastic efficacy, as blocking ROS production by NAC co-treatment abrogates the *in vitro* cytotoxic effects of chaetocin (e.g., Figure 2A) without directly reducing chaetocin (Tibodeau *et al*, 2009). Interestingly, although chaetocin induces mitochondrial membrane depolarisation and apoptosis, chaetocin-induced ROS and cell death appear nonetheless not dependant upon these phenomena (Figure 4). Likewise, although chaetocin induces morphological changes, including vacuole formation, that might be interpreted as associated with autophagy, autophagy also appears not to contribute to chaetocin-induced cytotoxicity, as autophagy inhibitors similarly do not block chaetocin-induced cell death (Figure 4). Considered collectively, our results indicate that chaetocin can kill cancer cells apparently by the induction of necrotic cell death, largely independent of reliance on intact PCD pathways. We interpret these results as quite promising and important as they suggest that chaetocin has potential to therefore be effective in killing cancer cells even harbouring defective PCD pathways that might otherwise render them resistant to other therapeutics.

In this regard, we hypothesise that the imposition of intracellular ROS by chaetocin by inhibition of thioredoxin reductase (Isham *et al*, 2007; Tibodeau *et al*, 2009) serves as a catastrophic cellular insult that can kill cells by necrosis even if apoptotic and autophagic pathways are disregulated. However, as the induction of cellular ROS might be expected to kill normal as well as malignant cells, the question arises as to why chaetocin might have anti-tumour selectivity, killing cancer cells whereas sparing normal cells—as we have clearly demonstrated to be the case in both myeloma (Isham *et al*, 2007), as well as also in the case of the SKOV3 solid tumour model (Figure 6). Our hypothesis in this regard is that cancer cells often display increased levels of basal cellular ROS relative to normal cells, in part because of the shift from energy production via the Krebs cycles instead of glycolysis (the ‘Warburg effect’) in cancer cells. In this scenario, because of the higher basal ROS levels in cancer cells, the imposition of cellular oxidative stress would more likely overwhelm ROS remediation systems in cancer cells than in normal cells, thereby potentially rendering cancer cells more sensitive than normal cells to redox-targeted agents like chaetocin (Fang *et al*, 2007). In this respect, our prior work (Tibodeau *et al*, 2009) indicates that chaetocin in fact serves to inhibit and disable thioredoxin reductase, a critical enzyme in the remediation of cellular ROS.

Also, potentially important are the results indicating that hypoxia and associated HIF-1α upregulation do not attenuate the *in vitro* antineoplastic effects of chaetocin (Figure 5C). These findings point to the possibility that the anticancer effects of chaetocin may remain largely intact in the hypoxic solid tumour microenvironment, a desirable property indeed with regard to a candidate solid tumour therapeutic. Of importance also is that although another group reported that functional HIF-1*α* is required for the antitumour effects of chaetocin in some systems (Lee *et al*, 2011), this did not seem to be the case in our experiments (e.g., Figure 5C).

Results indicating that chaetocin may not only target tumour cells directly but also indirectly by its antiangiogentic effects on tumour microvasculature (Figure 6) are also noteworthy. In particular, we found chaetocin to attenuate tumour-associated vascularity *in vivo* and to have potential to act directly in the tumour microenvironment, as chaetocin inhibits HuVEC proliferation (Figure 6D and E). These findings intriguingly suggest that chaetocin not only has direct effects on tumour but potentially also additional effects on the tumour microenvironment, that may combine together to produce observed *in vivo* efficacy.

Although our studies go a long way in clarifying the effects of chaetocin on solid tumour cells *in vitro*, questions still remain related to its actions in the tumour microenvironment and the extent to which these extra-tumour effects may contribute to observed *in vivo* activity. In particular, the effects of chaetocin in the setting of tumour hypoxia require more clarification. Although our experiments failed to demonstrate any effects of chaetocin on phospho-VEGFR-1 levels *in vivo* (suggesting that VEGF activity in treated and untreated SKOV3 tumours was similar), the results of others indicate instead that disruption of HIF-1*α* signalling may nevertheless have an important role in the *in vivo* effects of chaetocin under at least some circumstances. In particular, Kung *et al* (2004) were the first to implicate thiodioxopiperazines as candidate inhibitors of HIF-1*α* signalling, discovering in an unbiased high-throughput screen that the structurally related thiodioxopiperazine chaetomin attenuates HIF-1*α*-directed transcription by disrupting HIF-1α/p300 binding interactions *in vivo* in a HepG2 xenograft model. While the studies reported herein were ongoing, Lee *et al* (2011) have additionally implicated intact HIF-1α signalling as critical to the effects of chaetocin itself in at least some circumstances, demonstrating that chaetocin inhibits tumour growth *in vivo* in a HIF-1*α*(+/+) fibrosarcoma xenograft model that is abrogated in a corresponding HIF-1*α*(−/−) model, hence implicating chaetocin-altered splicing of HIF-1*α* pre-mRNA as contributory to *in vivo* effects in this model system (Iwasa *et al*, 2010).

Seemingly in contrast, our results instead demonstrate that chaetocin is cytotoxic in many tumour cell lines under normoxic *in vitro* conditions where HIF-1α is not upregulated or detectable (e.g., Figures 2A and 5C), clearly indicating that HIF-1*α* is not required for its *in vitro* cytotoxicity. We have established instead that, at least *in vitro*, chaetocin-induced cytotoxicity is dependant upon the ability of chaetocin to potently induce ROS (e.g., Figure 2), apparently via competitive inhibition of thioredoxin reductase (Tibodeau *et al*, 2009). Other contributors to *in vivo* effects undoubtedly also exist. In particular, the inhibition of HuVEC proliferation by chaetocin (Figure 6D and E) suggests that chaetocin may additionally act directly on endothelial cells in the tumour microenvironment. Furthermore, chaetocin also inhibits histone methyltransferase SU(VAR)3–9 (Greiner *et al*, 2005). It seems therefore likely that the multiple effects of chaetocin may combine to contribute to its *in vivo* activity, perhaps varying dependant upon tumour and tumour microenvironment contexts and accounting for apparently disparate observations in different model systems.

Collectively, chaetocin appears to represent an interesting and promising compound for further preclinical and clinical development in both myeloma and solid tumours. Its ability to kill cancer cells even in the presence of (i) inhibitors of apoptosis or autophagy, (ii) metabolically inactive mitochondria, or (iii) hypoxia are particularly intriguing, as is its potential to target not only just tumour cells but potentially also the tumour microenvironment by attenuation of endothelial cell proliferation.

## Figures and Tables

**Table 1:**
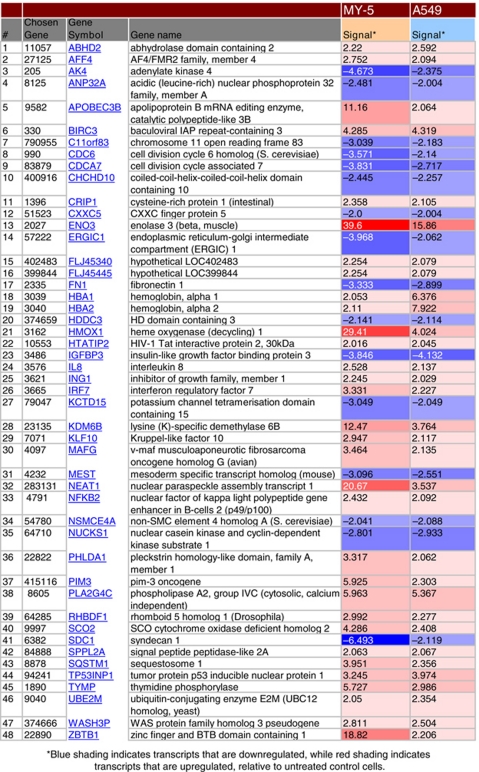
Common transcripts altered by chaetocin in both MY-5 and A549 cells
